# Applying the concept of liquid biopsy to monitor the microbial biodiversity of marine coastal ecosystems

**DOI:** 10.1038/s43705-022-00145-0

**Published:** 2022-07-27

**Authors:** Sophia Ferchiou, France Caza, Philippine Granger Joly de Boissel, Richard Villemur, Yves St-Pierre

**Affiliations:** INRS-Centre Armand-Frappier Santé Biotechnologie, Laval, Québec H7V 1B7 Canada

**Keywords:** Virology, Ecology

## Abstract

Liquid biopsy (LB) is a concept that is rapidly gaining ground in the biomedical field. Its concept is largely based on the detection of circulating cell-free DNA (ccfDNA) fragments that are mostly released as small fragments following cell death in various tissues. A small percentage of these fragments are from foreign (nonself) tissues or organisms. In the present work, we applied this concept to mussels, a sentinel species known for its high filtration capacity of seawater. We exploited the capacity of mussels to be used as natural filters to capture environmental DNA fragments of different origins to provide information on the biodiversity of marine coastal ecosystems. Our results showed that hemolymph of mussels contains DNA fragments that varied considerably in size, ranging from 1 to 5 kb. Shotgun sequencing revealed that a significant amount of DNA fragments had a nonself microbial origin. Among these, we found DNA fragments derived from bacteria, archaea, and viruses, including viruses known to infect a variety of hosts that commonly populate coastal marine ecosystems. Taken together, our study shows that the concept of LB applied to mussels provides a rich and yet unexplored source of knowledge regarding the microbial biodiversity of a marine coastal ecosystem.

## Introduction

Climate change (CC) impacts on the biodiversity of marine ecosystems are a rapidly evolving field of research. Global warming not only induces important physiological stress but also pushes the evolutionary limit of thermal tolerance of marine organisms, affecting the habitat of several species and pushing them to find more favorable conditions [1, 2]. In addition to its impact on the biodiversity of metazoans, CC also disrupts the delicate balance of host-microbe interactions. Such microbial dysbiosis is a major threat to marine ecosystems as it makes marine life more susceptible to infectious pathogens [3, 4]. CC is believed to play an important role in mass mortality events, a major concern for the management of marine ecosystems worldwide [5, 6]. This is an important issue given the economic, ecological, and nutritional impacts of many marine species. This is particularly true for bivalves found in polar regions where the effects of CC are more immediate and severe [6, 7]. In fact, bivalves, such as *Mytilus* spp., have been extensively used for monitoring the impact of CC in marine ecosystems. Not surprisingly, a relatively large number of biomarkers have been developed to monitor their health status, often using a two-tier approach that includes functional biomarkers based on enzymatic activities or cellular functions, such as cell viability and phagocytic activity [8]. These approaches also include measuring concentrations of specific stress indicators that accumulate in their soft tissues following uptake of high amounts of seawater. However, the high filtering capacities and the semi-open circulatory system of bivalves offer an opportunity to develop novel hemolymphatic biomarkers that exploit the concept of liquid biopsy (LB), a simple and minimally invasive approach used by clinicians for patient management based on a simple sample of blood [9, 10]. Although several types of circulating molecules can be detected in human LB, the concept is largely based on DNA sequencing analysis of circulating cell-free DNA (ccfDNA) fragments in plasma. In fact, the existence of DNA circulating in human plasma has been known since the middle of the 20th century [11], but it is only in recent years that the advent of high-throughput sequencing methods has led to clinical diagnostics based on ccfDNA. The presence of these circulating DNA fragments results in part from a passive release of genomic DNA (nuclear and mitochondrial) following cell death. In healthy individuals, the concentration of ccfDNA is normally low (<10 ng/mL) but can be increased by 5–10 times in patients suffering from various pathologies or subjected to stress, resulting in tissue damage. The size of ccfDNA fragments varies considerably but generally range from 150 to 200 bp [12]. Analysis of ccfDNA of *self-origin*, i.e., derived from host normal or transformed cells can be used to detect genetic and epigenetic alterations present in nuclear and/or mitochondrial genomes, thereby helping clinicians choose among specific molecularly targeted therapies [13]. However, ccfDNA can be derived from a *nonself* origin, such as ccfDNA derived from fetal cells during pregnancy or from transplanted organs [14–17]. ccfDNA is also an important source of information for detecting the presence of nucleic acids from infectious agents (*nonself*), thus making it possible to noninvasively detect a wide range of infections that are not identified by blood culture, avoiding invasive biopsy of infected tissues [18]. Recent studies have indeed shown that human blood contains a rich source of information for the identification of viral and bacterial pathogens and that ~1% of ccfDNA found in human plasma has a nonself origin [19]. These studies suggest that it is possible to assess the biodiversity of the circulating microbiome of an organism from the analysis of ccfDNA. Until very recently, however, this concept has exclusively been applied and studied in humans and, to a lesser extent, to other vertebrates [20, 21].

In the present work, we have taken advantage of the potential of LB to analyze the ccfDNA of *Aulacomya atra*, a southern species of mussels commonly found in the sub-Antarctic Kerguelen Archipelago, a group of islands located at the top of a large plateau that was built by volcanic eruptions 35 million years ago. Using an in vitro experimental system, we found that DNA fragments present in seawater are rapidly taken up by mussels and gain access to the hemolymphatic compartments. Shotgun sequencing showed that hemolymphatic ccfDNA of mussels contains DNA fragments of both self and nonself origin and included symbiotic bacteria as well as DNA fragments derived from biological communities that are typical of cold volcanic marine coastal ecosystems. Hemolymphatic ccfDNA also contained viral sequences derived from viruses with distinct host ranges. We also found DNA fragments derived from metazoans, such as bony fish, anemones, algae, and insects. Taken together, our study demonstrates that the concept of LB can be successfully applied to marine invertebrates to access a rich genomic reservoir within a marine ecosystem.

## Materials and methods

### Mussel collection

Adult specimens (55–70 mm length) of *Mytilus platensis* (*M. platensis*) and *Aulacomya atra* (*A. atra*) were collected on the intertidal rocky shore of Port-aux-Français (049°21.235S, 070°13.490E) at Kerguelen Islands in December 2018. Other adult blue mussels (*Mytilus spp.)* were obtained from a commercial supplier (PEI Mussel King Inc., Prince Edward Islands, Canada) and placed in a temperature-controlled (4 °C) aerated tank containing 10–20 L of 32‰ artificial saline water (Reef Crystal artificial marine salt, Instant Ocean, VA, USA). For each experiment, individual shell lengths and weights were measured.

### Circulating cell-free DNA extraction

A free and open access protocol for the procedure is available online (10.17504/protocols.io.81wgb6z9olpk/v1). Briefly, hemolymphatic LBs were collected from the abductor muscle as described [22]. The hemolymph was clarified by centrifugation at 1200 × *g* for 3 min, and the supernatant was frozen (−20 °C) until use. To isolate and purify ccfDNA, samples (1.5–2.0 mL) were thawed and processed using the NucleoSnap cfDNA kit (Macherey-Nagel, Bethlehen, PA) according to the manufacturer’s instructions. The ccfDNA was stored at −80 °C until further analysis. In some experiments, ccfDNA was extracted and purified using the QIAamp DNA Investigator Kit (QIAGEN, Toronto, ON, Canada). Purified DNA was quantified by standard PicoGreen assay. The fragment distribution of the extracted ccfDNA was analyzed by capillary electrophoresis with an Agilent 2100 Bioanalyzer (Agilent Technologies Inc., Santa Clara, CA) using a High Sensitivity DNA kit. The assay was performed according to the manufacturer’s instructions using 1 μL of ccfDNA sample.

### Sequence analysis pipeline

To sequence the hemolymphatic ccfDNA fragments, shotgun libraries were prepared by Génome Québec (Montreal, Quebec, Canada) using the Illumina DNA Mix set for Illumina MiSeq PE75. Standard adapters (BioO) were used. Raw data files are available on the NCBI Sequence Read Archive (SRR8924808 and SRR8924809). Basic read quality was assessed using FastQC [23]. The adapters and low-quality reads were trimmed with Trimmomatic [24]. The paired-end shotgun reads were merged into longer single reads with FLASH with a minimum overlap of 20 bp to avoid mismatches [25]. Merged reads were annotated with BLASTN using a bivalve NCBI Taxonomy database (*e* value < 1e^−3^ and 90% homology), and masking of low-complexity sequences was performed using DUST [26]. Reads were divided into two groups: those that were related to bivalve sequences (here named *self* reads) and those not (*nonself* reads). Both groups were assembled separately with MEGAHIT to generate contigs [27]. In parallel, the taxonomic distribution of microbiome nonself reads was classified with Kraken2 [28] and represented graphically with a Krona pie chart on Galaxy [29, 30]. Optimal kmers were determined from our preliminary experiments as kmers-59. Self contigs were then identified by alignment with BLASTN (bivalve NCBI database, *e* value < 1e^−10^ and 60% homology) for a final annotation. In parallel, nonself group contigs were annotated with BLASTN (nt NCBI database, *e* value < 1e^−10^ and 60% homology). BLASTX was also conducted on nonself contigs using the nr and RefSeq protein NCBI databases (*e* value < 1e^−10^ and 60% homology). A pool of BLASTN and BLASTX from nonself contigs represented the final set of contigs (see supplementary file).

### PCR amplification

The primers used for PCR are listed in Table S1. Taq DNA polymerase (Bio Basic Canada, Markham, ON) was used to amplify ccfDNA targeted genes. The following reaction conditions were employed: denaturation at 95 °C for 3 min; 35 cycles of 95 °C for 1 min, the prescribed annealing temperature for 1 min and elongation at 72 °C for 1 min; and a final 72 °C for 10 min. PCR products were separated by electrophoresis in agarose gels (1.5%) containing SYBR^TM^ Safe DNA Gel Stain (Invitrogen, Burlington, ON, Canada) at 95 V.

### DNA uptake by mussels

Mussels (*Mytilus* spp.*)* were acclimated in 500 mL of oxygenated seawater (32 PSU) at 4 °C for 24 h. Plasmidic DNA containing an insert encoding the cDNA sequence of the human galectin-7 gene (NCBI Accession number L07769) was added to the tank at a final concentration of 190 pg/µL. Controls included mussels incubated under the same conditions without the addition of DNA. A third control tank contained DNA without mussels. To track the quality of DNA in seawater, samples (20 µL; triplicates) of seawater were withdrawn from each tank at the indicated times. To track plasmid DNA in mussels, LBs were collected at the indicated times and analyzed by qPCR and ddPCR. Given the high salt levels in seawater, the aliquots were diluted in PCR quality water (1:10) before all PCR analyses.

### Digital droplet PCR

Digital droplet PCR (ddPCR) was performed using the QX200 BioRad (Mississaugua, ON, Canada) protocol. Optimal temperatures were established using temperature curves (Table S1). Droplets were generated with a QX200 droplet generator (BioRad). ddPCR was performed as follows: 95 °C for 5 min; 50 cycles of 95 °C for 30 s and the indicated annealing temperature for 1 min and 72 °C for 30 s; 4 °C for 5 min; and 90 °C for 5 min. Droplet number and positive reactions (copies/µL) were measured with a QX200 droplet reader (BioRad). Samples with fewer than 10,000 droplets were rejected. No template control was carried out on each run of ddPCR.

### Real-time qPCR

qPCR was performed using Rotor-Gene^®^ 3000 (Corbett Research, Sydney, Australia) with *LGALS7*-specific primers. All qPCRs were performed in 20 μL with the QuantiFast SYBR Green PCR Kit (QIAGEN). The qPCRs were initiated with a 15-min incubation at 95 °C followed by 40 cycles of 95 °C for 10 s and 60 °C for 60 s with a single acquisition. A melting curve was generated at the end of the qPCR using 95 °C for 5 s, 65 °C for 60 s and 97  °C with continuous acquisition. Each qPCR was performed in triplicate, and no template controls were included.

## Results

### Uptake of DNA by mussels

**Fig. 1:**
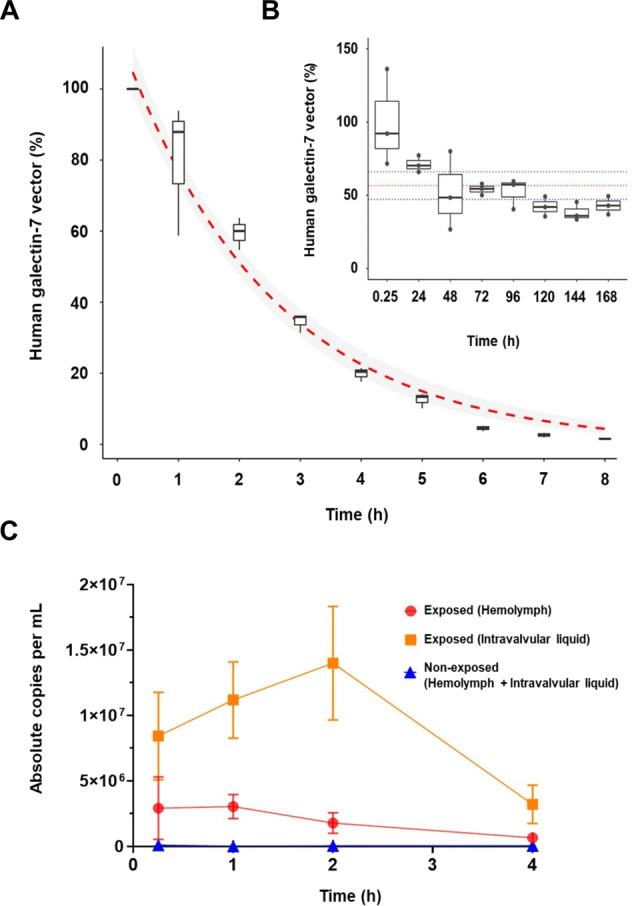
Experimental accumulation of nonself in mussels. Relative concentration of plasmidic DNA in seawater in presence (**A**) or absence (**B**) of mussels as measured by ddPCR. In **A**, the results are expressed as percentages and the limits of the box represent the 75th and 25th percentiles. A fitted logarithmic curve is represented in red with a gray shade area that represents the 95% confidence interval. In **B**, the red line indicates the mean value and blue lines represent the 95% confidence interval of the concentrations. **C** Accumulation of the plasmidic DNA in the hemolymph and intravalvular fluid of mussels at different time post-addition of plasmidic DNA. The results are shown as absolute number of copies/mL detected (±SE).

Because mussels are known for their high filtration rate capacity, we first studied whether they can filter and retain DNA fragments present in seawater. We were also interested in whether these fragments accumulate in their semi-open hemolymphatic system. We addressed this issue experimentally by tracking the fate of soluble DNA fragments added to aquariums containing blue mussels. To facilitate the tracking of the DNA fragments, we used foreign (nonself) plasmid DNA containing the human galectin-7 gene. Tracking of plasmidic DNA fragments in seawater and mussels was followed by ddPCR. Our results showed that although the amount of DNA fragments remained relatively stable over time (up to 7 days) in seawater in the absence of mussels, the levels almost completely disappeared within 8 h in the presence of mussels (Fig. 1a, b). Exogenous DNA fragments were readily detectable within 15 min in both intravalvular fluids and hemolymph (Fig. 1c). These fragments were still detected up to 4 h postexposure. Such filtering activity for DNA fragments is comparable to that reported for filtration of bacteria and algae [31]. These results suggest that mussels can filter and accumulate exogenous DNA in their fluidic compartments.

### Hemolymphatic ccfDNA in mussels

**Fig. 2:**
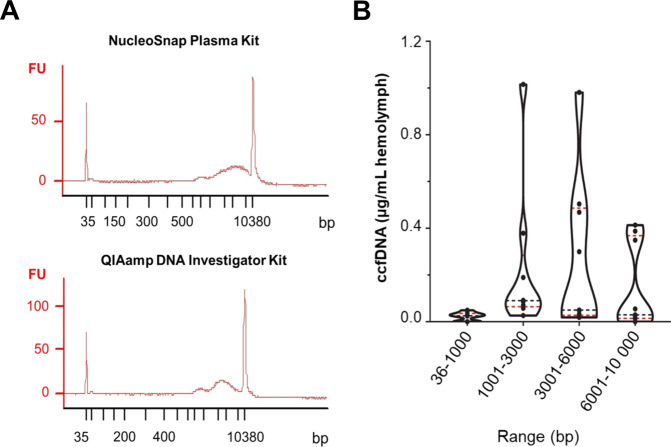
Fragment size distribution of hemolymphatic ccfDNA in mussels. **A** Representative electropherograms of the hemolymphatic ccfDNA of *Mytilus sp*. extracted with NucleoSnap Plasma Kit (above) and QIAamp DNA Investigator Kit. **B** Violin plots showing the distribution of the hemolymphatic ccfDNA concentrations (±SE) in mussels. Black and red lines represent the median and the first and third quartiles respectively.

We next studied the origin of ccfDNA in mussels collected in a mussel bed at Kerguelen Islands, a remote group of islands with limited anthropogenic impact. For this purpose, hemolymphatic ccfDNA from mussels was isolated and purified using methods that are commonly employed for purification of human ccfDNA [32, 33]. We found that the mean hemolymphatic ccfDNA concentrations in mussels were in the range of low micrograms per mL of hemolymph (see Table S2, supplementary information). Such a range of concentrations is significantly greater than that found in healthy humans (low nanograms per mL); however, ccfDNA levels in cancer patients can reach several micrograms per mL in rare cases [34, 35]. Analysis of the size distribution of hemolymphatic ccfDNA showed that these fragments varied considerably in size, ranging from 1000 bp to 5000 bp (Fig. 2). Similar results were obtained using the silica-based QIAamp Investigator kit, a method commonly used in forensic science to rapidly isolate and purify genomic DNA from samples at low DNA concentrations, including ccfDNA [36].

### Shotgun sequencing of hemolymphatic ccfDNA

In humans and primates, ~1% of ccfDNA has a nonself origin [21, 37]. Given the semi-open circulatory system of bivalves, the microorganism-rich seawater, and the size profile of mussel ccfDNA, we hypothesized that hemolymphatic ccfDNA of mussels is likely to contain a rich and diverse reservoir of microbial DNA. To test this hypothesis, we performed shotgun sequencing of hemolymphatic ccfDNA of *Aulacomya atra* specimens collected at Kerguelen Islands, generating more than 10 million reads, of which 97.6% passed quality control. Reads were then classified based on self and nonself origins using BLASTN and the NCBI bivalve database (Fig. S1, supplementary information).

### ccfDNA of self-origin

**Fig. 3:**
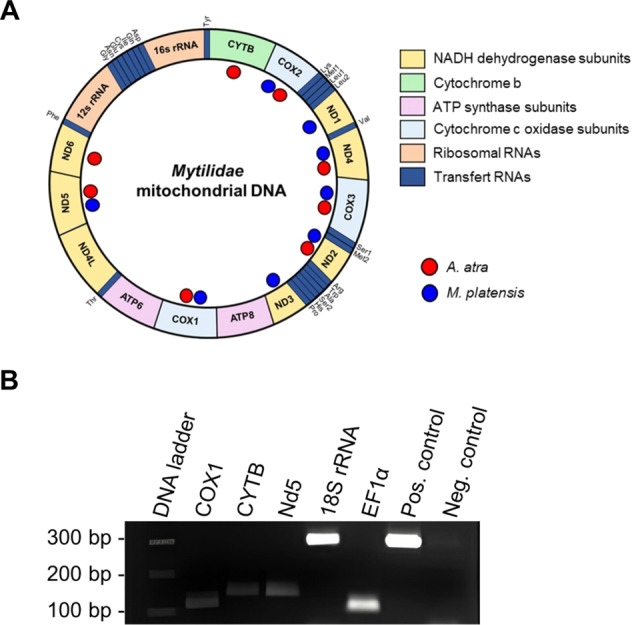
Validation of DNA fragments of self-origin. **A** Presence of different mitochondrial genes in the hemolymph of *A. atra* (red points – Accession: SRX5705969) and *M. platensis* (blue points – Accession: SRX5705968) amplified by PCR. Figure was adapted from Breton *et al*., 2011 **B** Amplifications of hemolymph supernatant from *A. atra* stored on FTA papers. PCR amplifications of HKG 18S rRNA, Elongation factor 1α (EF1α) and mitochondrial genes Cytochrome b (CYTB), Cytochrome c oxidase subunit 1 (COX1), and NADH dehydrogenase subunit 5 (Nd5) were carried out with a 3 mm punch directly added into the PCR tube containing the PCR mix.

In humans, both nuclear and mitochondrial DNA can be released in circulation [38]. In the present study, however, it was not possible to characterize in detail the nuclear genomic DNA of mussels given that the genome of *A. atra* has not yet been sequenced and reported. However, we were able to identify a number of ccfDNA fragments of self-origin using bivalve libraries (Fig. S2, supplementary information). We also confirmed the presence of DNA fragments of self-origin using targeted PCR amplification of those *A. atra* genes that have been sequenced (Fig. 3). Similarly, given that the mitochondrial genome of *A. atra* was available in public databases, it was possible to find evidence of mitochondrial ccfDNA fragments in the hemolymph of *A. atra*. The presence of mitochondrial DNA fragments was confirmed by PCR amplification (Fig. 3).

### Bacterial microbiome analysis of ccfDNA using Kraken2

**Fig. 4:**
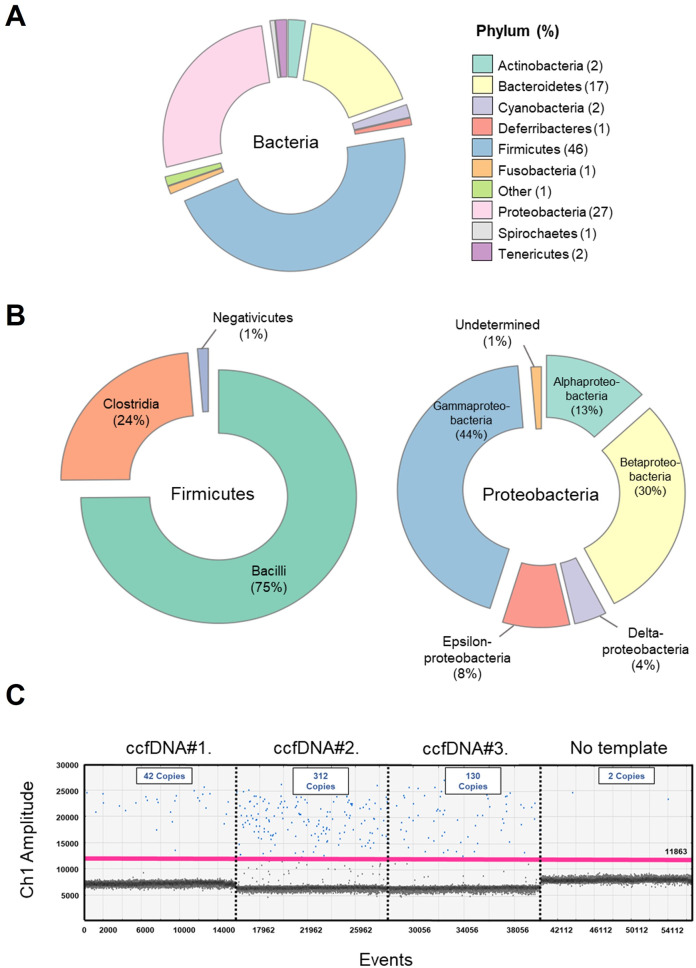
ccfDNA of bacterial origin. **A** Relative abundance at the phylum-level. **B** Microbial diversity of the two top phyla (Firmicutes and Proteobacteria). **C** Representative ddPCR amplification of *Vibrio* spp. 16S rRNA gene fragment (blue color) in three hemolymphs of *A. atra*.

**Fig. 5:**
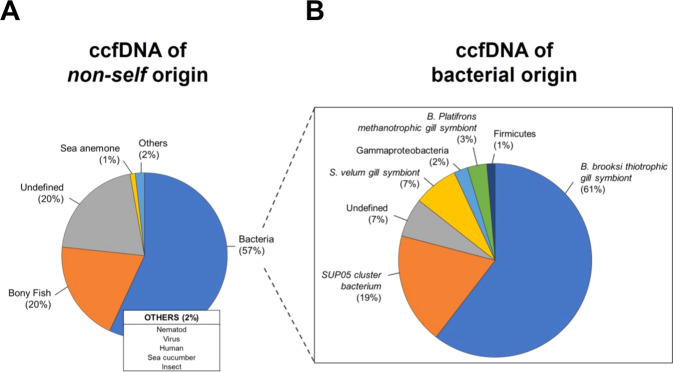
ccfDNA of various origins as identified using BLASTN and BLASTX. A total of 482 assembled contigs were analyzed. **A** Overall taxonomic distribution profile of metagenomic contig annotation (prokaryotes and eukaryotes). **B** Detailed distribution of bacterial DNA fragments identified using BLASTN and BLASTX.

Given the rich microbial content of marine seawater, we initially focused on the characterization of hemolymphatic microbial DNA sequences. For this, we used two distinct strategies. The first strategy employed Kraken2, an algorithm-based sequence classification program, which allows identification of microbial sequences with an accuracy comparable to BLAST and other tools [28]. Greater than 6719 reads were identified to be of bacterial origin, whereas 124 and 64 were of archaeal and viral origins, respectively (Fig. 4). The most prevalent bacterial DNA fragments originated from *Firmicutes* (46%), *Proteobacteria* (27%), and *Bacteroides* (17%) (Fig. 4a). This distribution was consistent with previous microbiome studies in marine blue mussels [39, 40]. *Gammaproteobacteria* were the dominant class of *Proteobacteria* (44%) and included many *Vibrionales* (Fig. 4b). The presence of DNA fragments of the *Vibrio* genus in hemolymphatic ccfDNA of *A. atra* was confirmed by ddPCR (Fig. 4c) [41]. To obtain more information on the bacterial origin of ccfDNA, a complementary approach was used (Figure S2, supplementary information). In this case, reads that overlapped were assembled as paired-end reads and were classified as of self (bivalves) or nonself origin using BLASTN and an *e* value of 1e^−3^ and a cutoff with >90% homology. Because the genome of *A. atra* has not yet been sequenced, we used a de novo assembly strategy with the MEGAHIT next-generation sequencing (NGS) assembler. A total of 147 188 contigs were identified as being of nonself (bivalve) origin. These contigs were then blasted using BLASTN and BLASTX using an *e* value of 1e^−10^. This strategy allowed us to identify 482 non-bivalve fragments present within the ccfDNA of *A. atra*. Greater than half (57%) of these DNA fragments were of bacterial origin with a majority from gill symbionts that included thiotrophic symbionts and from *Solemya velum* gill symbionts (Fig. 5).

### Archeal microbiome

**Fig. 6:**
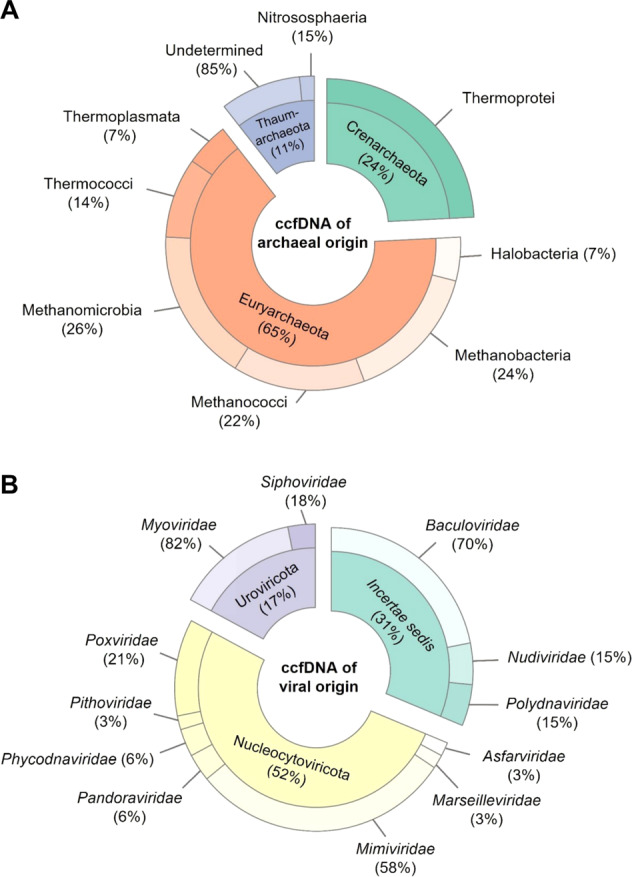
ccfDNA fragments of archaeal and viral origins. Classification of nonself reads using the Kraken taxonomic classification system.

Kraken2 analysis also showed that ccfDNA from mussels contained DNA fragments derived from Archaea, including from *Euryarchaeota* (65%), *Crenarchaeota* (24%), and *Thaurmarcheota* (11%) (Fig. 6a). The presence of DNA fragments derived from *Euryarchaeota* and *Crenarchaeota*, which have previously been found in the microbe assemblage of *Mytilus californicus*, may not be surprising [42]. Although *Euryarchaeota* have been commonly associated with extreme environments, it is now recognized that both *Euryarcheota* and *Crenarcheota* are among the most abundant prokaryotes in oceanic low-temperature environments [43, 44]. The presence of methanothopic microorganisms in mussels is not unexpected given recent reports of widespread methane seeps escaping from the seafloor of the Kerguelen Plateau [45] and the potential microbial methane production observed in coastal areas of Kerguelen Islands [46].

### Circulating virome

Our attention was then turned to reads derived from DNA viruses. To our knowledge, this is the first untargeted study of the viral content in mussels. As expected, we found DNA fragments derived from bacteriophages (*Caudovirale*s) (Fig. 6b). However, the most prevalent viral DNA originated from the phylum *Nucleocytoviricota*, which is also known as nucleocytoplasmic large DNA viruses (NCLDVs) and harbors the largest genomes among any viruses. Among this phylum, a majority of DNA sequences were derived from *Mimiviridae* (58%) and *Poxviridae* (21%), the natural hosts of which include vertebrates and arthropods, and a lesser extent of these DNA sequences were derived from *Phycodnaviridae*, which are known to infect marine eukaryotic algae. Sequences from *Pandoravirus*, a genus of giant virus with the largest genome size of any known viral genus, were also obtained. Interestingly, the range of hosts known to be infected by viruses that we identified through sequencing of hemolymphatic ccfDNA was relatively large (Figure S3, supplementary information). It includes viruses known to infect insects, such as *Baculoviridae* and *Iridoviridae*, as well as those known to infect amoebae, algae, and vertebrates. We also found sequences that matched genomic sequences of *Pithovirus sibericum*. Pithoviruses (aka “Zombie viruses”) were first isolated from a 30,000-year-old permafrost layer in Siberia [47]. Our findings are thus consistent with a previous report showing that modern species of these viruses have not gone all extinct [48], and these viruses may be found in distant subarctic marine ecosystems.

### Detection of metazoan-derived nonself ccfDNA sequences

We finally examined whether we could find DNA fragments originating from other metazoans. A total of 482 nonself contigs were identified using BLASTN and BLASTX performed with nt, nr and RefSeq libraries (genomes and proteins). Our results showed that metazoan nonself ccfDNA fragments were dominated by DNA from bony fish (Fig. 5). DNA fragments from insects and other species were also found. A relatively large percentage of DNA fragments were not identified possibly because a large number of marine species are underrepresented in genomic databases compared to terrestrial species [49].

## Discussion

In the present work, we applied the concept of LB to mussels, arguing that shotgun sequencing of hemolymphatic ccfDNA could provide insights into the constituents of a marine coastal ecosystem. More specifically, we showed that 1) the hemolymph of mussels contains a relatively high concentration (at the microgram level) of relatively large (~1–5 kb) circulating DNA fragments; 2) these DNA fragments are of both a self and nonself origin; 3) among the nonself origins of these DNA fragments, we found bacterial, archaeal and viral DNA as well as DNA from other metazoans; and 4) the accumulation of these hemolymphatic nonself ccfDNA fragments in the hemolymph is rapid and favored by the intrinsic filtration activity of mussels. Taken together, our study shows that the concept of LB, which has mostly been applied in the biomedical field to date, encodes a rich and yet unexplored source of knowledge that could be used to better understand the interactions between sentinel species and their environment.

In addition to primates, isolation of ccfDNA has been reported in mammals, including mice, dogs, cats, and horses [50–52]. To our knowledge, however, our study is the first to report the detection and sequencing of ccfDNA of a marine species with an open circulatory system. This anatomical feature and the filtering capacity of mussels probably explains, at least in part, the distinct size profile of circulating DNA fragments when compared to other species. In humans, most DNA fragments that circulate into the bloodstream are small fragments ranging between 150 and 200 bp with a maximum peak at 167 bp [34, 53]. A smaller but significant proportion of DNA fragments falls between 300 and 500 bp, and ~5% of DNA fragments are longer than 900 bp [54]. This size distribution is explained by the fact that the major source of ccfDNA in plasma originates from cell death, either due to apoptosis or following necrosis of circulating hematopoietic cells in healthy individuals or tumor cell apoptosis in cancer patients (referred to as circulating tumor DNA, ctDNA). The size distribution of hemolymphatic ccfDNA we found in mussels, which ranges from 1000 to 5000 bp, suggests that the ccfDNA of mussels has a different origin. This is a logical hypothesis given that mussels have a semi-open vascular system and live in a marine aquatic environment that contains high concentrations of genomic DNA derived from microorganisms. In fact, our laboratory experiments using foreign DNA suggest that mussels accumulate DNA fragments present in seawater, at least for several hours after which they are either degraded, and/or released, and/or stored in different tissues following cellular uptake. Using the intravalvular compartment would reduce the ccfDNA from self origin, but also from nonself origin considering the rarity of (prokaryotic and eukaryotic) cells. Considering the importance of innate immunity in bivalves and the high numbers of circulating phagocytes, we further hypothesize that even nonself ccfDNA is enriched by the circulating phagocytes which accumulate foreign DNA upon phagocytosis of microorganisms and/or cell debris. Taken together, our findings suggest that hemolymphatic ccfDNA in bivalves are a unique reservoir of molecular information and reinforces their status as sentinel species.

Our data showed that sequencing and analysis of bacterial-derived hemolymphatic ccfDNA fragments can provide critical information on the bacterial flora of the host and bacteria present in the surrounding marine ecosystem. The shotgun sequencing approach revealed sequences from the gill symbiont bacteria of *A. atra* that would otherwise have been missed if the common 16S rRNA identification method was employed partly due to a bias in the reference library. In fact, our data using LB collected from *M. platensis* in the same mussel bed at Kerguelen showed that both mussel species had a similar composition of their gill-associated bacterial symbiont (Figure S4, supplementary information). Such similarity for both genetically distinct mussels possibly reflects the composition of the bacterial community in the cold, sulfidic and volcanic sediment of Kerguelen [55–58]. A higher proportion of sulfur-reducing microorganisms is well described in bioturbated coastal zones [59], such as the coast of Port-aux-Français, where mussels were collected. Another possibility is that the mussel symbiont flora can be influenced by horizontal transmission [60, 61]. More studies will be required to determine the correlation between the marine environment, the ocean floor surface and the mussel symbiotic bacterial composition. These studies are currently underway.

The length and concentration of hemolymphatic ccfDNA, the ease of its purification and its high quality that allows for rapid shotgun sequencing are among the many benefits of using ccfDNA of mussels to assess the biodiversity of marine coastal ecosystems. This approach is particularly effective for characterizing viral communities (virome) within a given ecosystem [62, 63]. In contrast to bacteria, archaea and eukaryotes, viral genomes do not harbor phylogenetically conserved genes, such as the 16S sequences. Our findings, showed that liquid biopsies from sentinel species like mussels can be used to identify a relatively large number of ccfDNA fragments of viruses known to infect hosts which commonly populate coastal marine ecosystems. This included viruses known to infect protists, arthropods, insects, plants, and bacterial viruses (i.e., bacteriophages). A similar distribution was found when we studied the virome of hemolymphatic ccfDNA of blue mussels (*M. platensis*) collected in the same mussel beds at Kerguelen (Table S2, supplementary information). Shotgun sequencing of ccfDNA is indeed a new approach that has gained momentum for studying the virome in humans or other species [21, 37, 64]. This approach is particularly useful for studying dsDNA viruses because not a single gene is conserved in all dsDNA viruses, which represent the most diverse and expansive Baltimore classes of viruses [65]. Although most of these viruses remain unclassified and likely include viruses in completely uncharted parts of the virus world [66], we found that the virome of both mussel *A. atra* and *M. platensis* species and the host range were similar between both species (see Fig. S3, supplementary information). Such similarity is not surprising, as it likely reflects the lack of selectivity during the uptake of DNA present in the surrounding environment. Future studies using purified RNA are currently needed to characterize the RNA virome.

In our study, we used a very stringent pipeline that was adapted from the work of Kowarsky and colleagues [37] who used a two-step removal of self ccfDNA before and after the assembly on its merged reads and contigs, thereby generating a large proportion of unmapped reads. Accordingly, we cannot rule out that a proportion of these unmapped reads can still be of self origin, most notably as we do not have a reference genome for this mussel species. We also used this pipeline because we were concerned by chimeras formed between self and nonself reads and the length of the reads generated by the Illumina MiSeq PE75s. Another reason for the large proportion of unmapped reads is that a large proportion of marine microorganisms, especially in such a remote area as Kerguelen, has not yet been annotated. We used the Illumina MiSeq PE75, assuming that the ccfDNA length fragments would be similar to human ccfDNA. For future studies, given our results showing that hemolymphatic ccfDNA has longer reads than that of humans and/or mammals, we would recommend sequencing platforms that are more adapted to longer ccfDNA fragments. This practice would greatly facilitate the identification of a higher number of reads, allowing deeper analyses. Obtaining a complete sequence of the nuclear genome of *A. atra*, which is not currently available, would also greatly facilitates the distinction between ccfDNA of self and nonself origin. Considering that our study was focused on the feasibility of applying the concept of liquid biopsy to mussels, we are hopeful that as future studies exploit this concept, new tools and pipelines will be developed to improve that potential of this method to study the microbial biodiversity of marine ecosystems.

As a noninvasive clinical biomarker, elevated levels of human plasmatic ccfDNA have been associated with several diseases, tissue damage and stress conditions [67–69]. This increase is attributed to the release of DNA fragments of self-origin upon tissue damage. We examined this issue using acute thermal stress where mussels were exposed for a short period of time at 30 °C. We performed this assay with three different species of mussels in three independent experiments. We did not however detect any variations in the ccfDNA levels following acute thermal stress (see Fig. S5, supplementary information). This finding is likely explained, at least in part, to the fact that mussels have a semi-open circulatory system and accumulate large concentrations of nonself DNA given their high filtering activity. Alternatively, mussels, such as many invertebrates, may be more tolerant to stress-induced tissue damage, limiting the release of ccfDNA in their hemolymph [70, 71].

To date, DNA analysis of biodiversity in aquatic ecosystems has mostly centered on environmental DNA (eDNA) metabarcoding. This approach, however, is often limited in terms of biodiversity analysis when using primers. The use of shotgun sequencing bypasses the PCR limitations and a biased selection of primer sets. In a sense, our approach is thus closer to the more recently used high-throughput eDNA shotgun sequencing methods that enable direct sequencing of fragmented DNA and analysis of basically all living organisms [72, 73]. However, there are a number of fundamental issues that distinguish LB from standard eDNA approaches. Of course, the major difference between eDNA and LB is the use of a natural filtering host. The use of marine species, such as sponges and bivalves (*Dresseina* spp.), as natural filter to study eDNA has been reported [74, 75]. The study on *Dreissena*, however, used tissular biopsy from which DNA was extracted. Analysis of ccfDNA from LB does not require tissular biopsy and specialized and occasionally costly equipment and logistics associated with eDNA or tissular biopsies. In fact, we have recently reported that ccfDNA from LB can be stored and analyzed on FTA support, bypassing the need for maintaining a cold chain, a major issue for studies in remote regions [76]. Extraction of ccfDNA from liquid biopsies is also simple and provides high-quality DNA for shotgun sequencing and PCR analysis. This is a major advantage considering some of the technical limitations associated with eDNA analysis [77]. The simplicity and low cost of the sampling method is also particularly well adapted for long-term monitoring surveillance programs. Another well-known feature of bivalves, in addition to their high filtering capacity, is the chemical mucopolysaccharidic composition of their mucus, which favors the uptake of viruses [78, 79]. This makes bivalves an ideal natural filter to characterize the biodiversity of a given aquatic ecosystem and the impacts of CC. Although the presence of DNA fragments from the host can be viewed as a limit to the approach compared to eDNA, the cost associated by the presence of such self ccfDNA is offset by the wealth of information that can be simultaneously obtained to study on the health status of the host. This includes the presence of viral sequences integrated in the host genome of the host. This is particularly important in the case of mussels given the existence of horizontally-transmitted leukemogenic retroviruses in bivalves [80, 81]. Another advantage of LB compared to eDNA is that it exploits the phagocytic activity of hemolymphatic circulating hemocytes which engulfed microorganisms (and their genome). Phagocytosis is the most fundamental role for hemocytes in bivalves [82]. Finally, the approach takes advantage of the high filtration capacity of mussels (which pumps an average of 1.5 L/h of seawater) and the bi-diurnal cycles, both of which increases the mixing of different layers of seawater columns, thereby allowing the capture of heterogenous eDNA [83, 84]. Analysis of ccfDNA from mussels is thus an interesting avenue considering their nutritional, economic and ecological impact. In a manner similar to the analysis of LB collected in humans, the approach further opens up the possibility of measuring genetic and epigenetic alterations of the host’s DNA in response to xenobiotics. For example, it is possible to envisage third-generation sequencing technologies to perform genome-wide analysis of methylation in ccfDNA of self-origin using nanopore sequencing. This process should be facilitated by the fact that the length of ccfDNA fragments of mussels is ideally compatible with long-read sequencing platforms that enable genome-wide analysis of DNA methylation from a single sequencing run without the need for chemical conversion [85, 86]. This is an interesting possibility because DNA methylation patterns have been shown to reflect responses to environmental stress and persist for many generations. It could thus provide valuable information on potential mechanisms regulating responses following exposure to climate change or pollutants [87]. The use of LB, however, is not without limitations. Needless to say, it requires the presence of sentinel species in the ecosystem. As mentioned above, the use of LB to assess the biodiversity of a given ecosystem also requires a stringent bioinformatic pipeline to take into account the presence of DNA fragments of self-origin. The other major challenge is the availability of reference genomes from marine species. Hopefully, initiatives, such as the marine mammal genome project and the recently established Fish10k project [88], will facilitate such analysis in the future. The application of the LB concept to marine filtering organisms is also compatible with recent advances in sequencing technologies, rendering it fully suited for the development of multiomics biomarkers to provide important information on the health status of marine habitats in response to environmental stress.

## Supplementary information

Supplementary information

## Data Availability

Genome sequencing data have been deposited in the NCBI Sequence Read Archive https://www.ncbi.nlm.nih.gov/sra/SRR8924808 under the Bioprojects SRR8924808.

## References

[1] Brierley AS, Kingsford MJ (2009). Impacts of climate change on marine organisms and ecosystems. Curr Biol.

[2] Gissi E, Manea E, Mazaris AD, Fraschetti S, Almpanidou V, Bevilacqua S (2021). A review of the combined effects of climate change and other local human stressors on the marine environment. Sci Total Environ.

[3] Carella F, Antuofermo E, Farina S, Salati F, Mandas D, Prado P (2020). In the wake of the ongoing mass mortality events: co-occurrence of Mycobacterium, Haplosporidium and other pathogens in Pinna nobilis collected in Italy and Spain (Mediterranean Sea). Front Mar Sci.

[4] Seuront L, Nicastro KR, Zardi GI, Goberville E (2019). Decreased thermal tolerance under recurrent heat stress conditions explains summer mass mortality of the blue mussel Mytilus edulis. Sci Rep.

[5] Fey SB, Siepielski AM, Nussle S, Cervantes-Yoshida K, Hwan JL, Huber ER (2015). Recent shifts in the occurrence, cause, and magnitude of animal mass mortality events. Proc Natl Acad Sci USA.

[6] Scarpa F, Sanna D, Azzena I, Mugetti D, Cerruti F, Hosseini S (2020). Multiple non-species-specific pathogens possibly triggered the mass mortality in Pinna nobilis. Life..

[7] Bradley M, Kutz SJ, Jenkins E, O’Hara TM (2005). The potential impact of climate change on infectious diseases of Arctic fauna. Int J Circumpolar Health.

[8] Beyer J, Green NW, Brooks S, Allan IJ, Ruus A, Gomes T (2017). Blue mussels (Mytilus edulis spp.) as sentinel organisms in coastal pollution monitoring: a review. Mar Environ Res.

[9] Siravegna G, Marsoni S, Siena S, Bardelli A (2017). Integrating liquid biopsies into the management of cancer. Nat Rev Clin Oncol.

[10] Wan JCM, Massie C, Garcia-Corbacho J, Mouliere F, Brenton JD, Caldas C (2017). Liquid biopsies come of age: towards implementation of circulating tumour DNA. Nat Rev Cancer.

[11] Mandel P, Metais P (1948). Nuclear acids in human blood plasma. Comptes Rendus Séances Soc Biol Filiales.

[12] Bronkhorst AJ, Ungerer V, Holdenrieder S (2019). The emerging role of cell-free DNA as a molecular marker for cancer management. Biomol Detect Quantif.

[13] Ignatiadis M, Sledge GW, Jeffrey SS (2021). Liquid biopsy enters the clinic - implementation issues and future challenges. Nat Rev Clin Oncol.

[14] Lo YM, Corbetta N, Chamberlain PF, Rai V, Sargent IL, Redman CW (1997). Presence of fetal DNA in maternal plasma and serum. Lancet..

[15] Moufarrej MN, Wong RJ, Shaw GM, Stevenson DK, Quake SR (2020). Investigating pregnancy and its complications using circulating cell-free RNA in women’s blood during gestation. Front Pediatr.

[16] Oellerich M, Sherwood K, Keown P, Schutz E, Beck J, Stegbauer J (2021). Liquid biopsies: donor-derived cell-free DNA for the detection of kidney allograft injury. Nat Rev Nephrol.

[17] Wong FC, Lo YM (2016). Prenatal diagnosis innovation: genome sequencing of maternal plasma. Annu Rev Med.

[18] Gu W, Deng X, Lee M, Sucu YD, Arevalo S, Stryke D (2021). Rapid pathogen detection by metagenomic next-generation sequencing of infected body fluids. Nat Med.

[19] Huang YF, Chen YJ, Fan TC, Chang NC, Chen YJ, Midha MK (2018). Analysis of microbial sequences in plasma cell-free DNA for early-onset breast cancer patients and healthy females. BMC Med Genom.

[20] Goggs R, Jeffery U, LeVine DN, Li RHL (2020). Neutrophil-extracellular traps, cell-free DNA, and immunothrombosis in companion animals: a review. Vet Pathol.

[21] Kowarsky M, De Vlaminck I, Okamoto J, Neff NF, LeBreton M, Nwobegabay J, et al. Cell-free DNA reveals potential zoonotic reservoirs in non-human primates. BioRxiv. 2018;481093.

[22] Caza F, Bernet E, Veyrier FJ, Betoulle S, St-Pierre Y (2020). Hemocytes released in seawater act as Trojan horses for spreading of bacterial infections in mussels. Sci Rep.

[23] Andrew S. FastQC: a quality control tool for high throughput sequence data. 2010. http://www.bioinformatics.babraham.ac.uk/projects/fastqc.

[24] Bolger AM, Lohse M, Usadel B (2014). Trimmomatic: a flexible trimmer for Illumina sequence data. Bioinformatics.

[25] Magoč T, Salzberg SL (2011). FLASH: fast length adjustment of short reads to improve genome assemblies. Bioinformatics..

[26] Morgulis A, Gertz EM, Schäffer AA, Agarwala R (2006). A fast and symmetric DUST implementation to mask low-complexity DNA sequences. Comput Biol.

[27] Li D, Liu CM, Luo R, Sadakane K, Lam TW (2015). MEGAHIT: an ultra-fast single-node solution for large and complex metagenomics assembly via succinct de Bruijn graph. Bioinformatics..

[28] Wood DE, Salzberg SL (2014). Kraken: ultrafast metagenomic sequence classification using exact alignments. Genome Biol.

[29] Cuccuru G, Orsini M, Pinna A, Sbardellati A, Soranzo N, Travaglione A (2014). Orione, a web-based framework for NGS analysis in microbiology. Bioinformatics..

[30] Ondov BD, Bergman NH, Phillippy AM (2011). Interactive metagenomic visualization in a Web browser. BMC Bioinform.

[31] Lüskow F, Riisgård H (2018). In situ filtration rates of blue mussels (Mytilus edulis) measured by an open-top chamber method. OJMS.

[32] Szpechcinski A, Struniawska R, Zaleska J, Chabowski M, Orlowski T, Roszkowski K (2008). Evaluation of fluorescence-based methods for total vs. amplifiable DNA quantification in plasma of lung cancer patients. J Physiol Pharmacol.

[33] Tissot C, Toffart AC, Villar S, Souquet PJ, Merle P, Moro-Sibilot D (2015). Circulating free DNA concentration is an independent prognostic biomarker in lung cancer. Eur Respir J.

[34] Kustanovich A, Schwartz R, Peretz T, Grinshpun A (2019). Life and death of circulating cell-free DNA. Cancer Biol Ther.

[35] Prouteau A, Denis JA, De Fornel P, Cadieu E, Derrien T, Kergal C (2021). Circulating tumor DNA is detectable in canine histiocytic sarcoma, oral malignant melanoma, and multicentric lymphoma. Sci Rep.

[36] Vandewoestyne M, Van Hoofstat D, Franssen A, Van Nieuwerburgh F, Deforce D (2013). Presence and potential of cell free DNA in different types of forensic samples. For Sci Int Genet.

[37] Kowarsky M, Camunas-Soler J, Kertesz M, De Vlaminck I, Koh W, Pan W (2017). Numerous uncharacterized and highly divergent microbes which colonize humans are revealed by circulating cell-free DNA. Proc Natl Acad Sci USA.

[38] Meddeb R, Dache ZAA, Thezenas S, Otandault A, Tanos R, Pastor B (2019). Quantifying circulating cell-free DNA in humans. Sci Rep.

[39] Li YF, Yang N, Liang X, Yoshida A, Osatomi K, Power D (2018). Elevated seawater temperatures decrease microbial diversity in the gut of Mytilus coruscus. Front Physiol.

[40] Musella M, Wathsala R, Tavella T, Rampelli S, Barone M, Palladino G (2020). Tissue-scale microbiota of the Mediterranean mussel (Mytilus galloprovincialis) and its relationship with the environment. Sci Total Environ.

[41] Thompson JR, Randa MA, Marcelino LA, Tomita-Mitchell A, Lim E, Polz MF (2004). Diversity and dynamics of a north atlantic coastal Vibrio community. Appl Environ Microbiol.

[42] Pfister CA, Meyer F, Antonopoulos DA (2010). Metagenomic profiling of a microbial assemblage associated with the California mussel: a node in networks of carbon and nitrogen cycling. PLoS One.

[43] Galand PE, Casamayor EO, Kirchman DL, Potvin M, Lovejoy C (2009). Unique archaeal assemblages in the Arctic Ocean unveiled by massively parallel tag sequencing. ISME J.

[44] Korzhenkov AA, Toshchakov SV, Bargiela R, Gibbard H, Ferrer M, Teplyuk AV (2019). Archaea dominate the microbial community in an ecosystem with low-to-moderate temperature and extreme acidity. Microbiome..

[45] Spain EA, Johnson SC, Hutton B, Whittaker JM, Lucieer V, Watson SJ (2020). Shallow seafloor gas emissions near Heard and McDonald Islands on the Kerguelen Plateau, southern Indian Ocean. Earth Space Sci..

[46] Farías L, Florez-Leiva L, Besoain V, Sarthou G, Fernández C (2015). Dissolved greenhouse gases (nitrous oxide and methane) associated with the naturally iron-fertilized Kerguelen region (KEOPS 2 cruise) in the Southern Ocean. Biogeosciences..

[47] Legendre M, Bartoli J, Shmakova L, Jeudy S, Labadie K, Adrait A (2014). Thirty-thousand-year-old distant relative of giant icosahedral DNA viruses with a pandoravirus morphology. Proc Natl Acad Sci USA.

[48] Levasseur A, Andreani J, Delerce J, Bou Khalil J, Robert C, La Scola B (2016). Comparison of a modern and fossil pithovirus reveals its genetic conservation and evolution. Genome Biol Evol.

[49] Kelley JL, Brown AP, Therkildsen NO, Foote AD (2016). The life aquatic: advances in marine vertebrate genomics. Nat Rev Genet.

[50] Colmer SF, Luethy D, Abraham M, Stefanovski D, Hurcombe SD (2021). Utility of cell-free DNA concentrations and illness severity scores to predict survival in critically ill neonatal foals. PLoS One.

[51] Rushton JG, Ertl R, Klein D, Tichy A, Nell B (2019). Circulating cell-free DNA does not harbour a diagnostic benefit in cats with feline diffuse iris melanomas. J Feline Med Surg.

[52] Tagawa M, Shimbo G, Inokuma H, Miyahara K (2019). Quantification of plasma cell-free DNA levels in dogs with various tumors. J Vet Diagn Investig.

[53] Shi J, Zhang R, Li J, Zhang R (2020). Size profile of cell-free DNA: a beacon guiding the practice and innovation of clinical testing. Theranostics..

[54] Fernando MR, Jiang C, Krzyzanowski GD, Ryan WL (2018). Analysis of human blood plasma cell-free DNA fragment size distribution using EvaGreen chemistry based droplet digital PCR assays. Clin Chim Acta.

[55] Findlay AJ (2016). Microbial impact on polysulfide dynamics in the environment. FEMS Microbiol Lett.

[56] Jørgensen BB, Findlay AJ, Pellerin A (2019). The biogeochemical sulfur cycle of marine sediments. Front Microbiol.

[57] Teske A, Brinkhoff T, Muyzer G, Moser DP, Rethmeier J, Jannasch HW (2000). Diversity of thiosulfate-oxidizing bacteria from marine sediments and hydrothermal vents. Appl Environ Microbiol.

[58] Zhang X, Du Z, Zheng R, Luan Z, Qi F, Cheng K (2017). Development of a new deep-sea hybrid Raman insertion probe and its application to the geochemistry of hydrothermal vent and cold seep fluids. Deep Sea Res Part I Oceanogr Res Pap.

[59] Egger M, Riedinger N, Mogollón JM, Jørgensen BB (2018). Global diffusive fluxes of methane in marine sediments. Nat Geosci.

[60] Ansorge R, Romano S, Sayavedra L, Kupczok A, Tegetmeyer HE, Dubilier N (2019). Functional diversity enables multiple symbiont strains to coexist in deep-sea mussels. Nat Microbiol.

[61] Russell SL, Pepper-Tunick E, Svedberg J, Byrne A, Ruelas Castillo J, Vollmers C (2020). Horizontal transmission and recombination maintain forever young bacterial symbiont genomes. PLoS Genet.

[62] Angly FE, Felts B, Breitbart M, Salamon P, Edwards RA, Carlson C (2006). The marine viromes of four oceanic regions. PLoS Biol.

[63] Li Z, Pan D, Wei G, Pi W, Zhang C, Wang JH (2021). Deep sea sediments associated with cold seeps are a subsurface reservoir of viral diversity. ISME J.

[64] Thongsripong P, Chandler JA, Kittayapong P, Wilcox BA, Kapan DD, Bennett SN (2021). Metagenomic shotgun sequencing reveals host species as an important driver of virome composition in mosquitoes. Sci Rep.

[65] Koonin EV, Krupovic M, Agol VI (2021). The Baltimore classification of viruses 50 years later: how does it stand in the light of virus evolution?. Microbiol Mol Biol Rev.

[66] Koonin EV, Dolja VV, Krupovic M, Varsani A, Wolf YI, Yutin N (2020). Global organization and proposed megataxonomy of the virus world. Microbiol Mol Biol Rev.

[67] Breitbach S, Tug S, Simon P (2012). Circulating cell-free DNA: an up-coming molecular marker in exercise physiology. Sports Med.

[68] Preissner KT, Herwald H (2017). Extracellular nucleic acids in immunity and cardiovascular responses: between alert and disease. Thromb Haemost.

[69] Schwarzenbach H (2013). Circulating nucleic acids as biomarkers in breast cancer. Breast Cancer Res.

[70] Murphy DJ (1983). Freezing resistance in intertidal invertebrates. Annu Rev Physiol.

[71] Robledo JAF, Yadavalli R, Allam B, Espinosa EP, Gerdol M, Greco S (2019). From the raw bar to the bench: bivalves as models for human health. Dev Comp Immunol.

[72] Cowart DA, Murphy KR, Cheng CC (2018). Metagenomic sequencing of environmental DNA reveals marine faunal assemblages from the West Antarctic Peninsula. Mar Genom..

[73] Parducci L, Bennett KD, Ficetola GF, Alsos IG, Suyama Y, Wood JR (2017). Ancient plant DNA in lake sediments. New Phytol.

[74] Mariani S, Baillie C, Giuliano C, Riesgo A (2019). Sponges as natural environmental DNA samplers. Curr Biol.

[75] Weber S, Brink L, Wörner M, Künzel S, Veith M, Teubner D, et al. Molecular diet analysis in zebra and quagga mussels (Dreissena spp.) and an assessment of the utility of aquatic filter feeders as biological eDNA filters. BioRxiv. 2021; 432951.

[76] Caza F, Joly de Boissel PG, Villemur R, Betoulle S, St-Pierre Y (2019). Liquid biopsies for omics-based analysis in sentinel mussels. Plos One.

[77] Hunter ME, Ferrante JA, Meigs-Friend G, Ulmer A (2019). Improving eDNA yield and inhibitor reduction through increased water volumes and multi-filter isolation techniques. Sci Rep.

[78] Burkhardt W, Calci KR (2000). Selective accumulation may account for shellfish-associated viral illness. Appl Environ Microbiol.

[79] Di Girolamo R, Liston J, Matches J (1977). Ionic bonding, the mechanism of viral uptake by shellfish mucus. Appl Environ Microbiol.

[80] Metzger MJ, Reinisch C, Sherry J, Goff SP (2015). Horizontal transmission of clonal cancer cells causes leukemia in soft-shell clams. Cell..

[81] Metzger MJ, Villalba A, Carballal MJ, Iglesias D, Sherry J, Reinisch C (2016). Widespread transmission of independent cancer lineages within multiple bivalve species. Nature..

[82] Canesi L, Gallo G, Gavioli M, Pruzzo C (2002). Bacteria–hemocyte interactions and phagocytosis in marine bivalves. Microsc Res Tech.

[83] Andruszkiewicz EA, Koseff JR, Fringer OB, Ouellette NT, Lowe AB, Edwards CA (2019). Modeling environmental DNA transport in the coastal ocean using Lagrangian particle tracking. Front Mar Sci.

[84] Wood ZT, Lacoursière-Roussel A, LeBlanc F, Trudel M, Kinnison MT, Garry McBrine C (2021). Spatial heterogeneity of eDNA transport improves stream assessment of threatened salmon presence, abundance, and location. Front Ecol Evol.

[85] Rand AC, Jain M, Eizenga JM, Musselman-Brown A, Olsen HE, Akeson M (2017). Mapping DNA methylation with high-throughput nanopore sequencing. Nat Methods.

[86] Simpson JT, Workman RE, Zuzarte PC, David M, Dursi LJ, Timp W (2017). Detecting DNA cytosine methylation using nanopore sequencing. Nat Methods.

[87] Cavalli G, Heard E (2019). Advances in epigenetics link genetics to the environment and disease. Nature..

[88] Fan G, Song Y, Yang L, Huang X, Zhang S, Zhang M (2020). Initial data release and announcement of the 10,000 Fish Genomes Project (Fish10K). Gigascience..

