# Supplementary material for: Applying the concept of liquid biopsy to monitor the microbial biodiversity of marine coastal ecosystems
Source: ISME Commun. 2022 Jul 27;2:61. doi: 10.1038/s43705-022-00145-0 (PMC9723566; doi:10.1038/s43705-022-00145-0)
Supplement: Supplementary file 1 — Supplementary information [file 43705_2022_145_MOESM1_ESM.pdf]

Applying the concept of liquid biopsy to monitor the microbial biodiversity of marine coastal ecosystems

Supplementary information

Sophia Ferchiou*, France Caza*, Philippine Granger Joly de Boissel, Richard Villemur and Yves St-Pierre

INRS-Centre Armand-Frappier Santé Biotechnologie, Laval, Québec, Canada, H7V 1B7.

* : These authors contributed equally to this work.

Corresponding Author : Yves St-Pierre, INRS-Centre Armand-Frappier Santé Biotechnologie, 531 Boul. Des Prairies, Laval, Québec, Canada, H7V 1B7.
E-mail: yves.st-pierre@inrs.ca

Supp. Figure 1

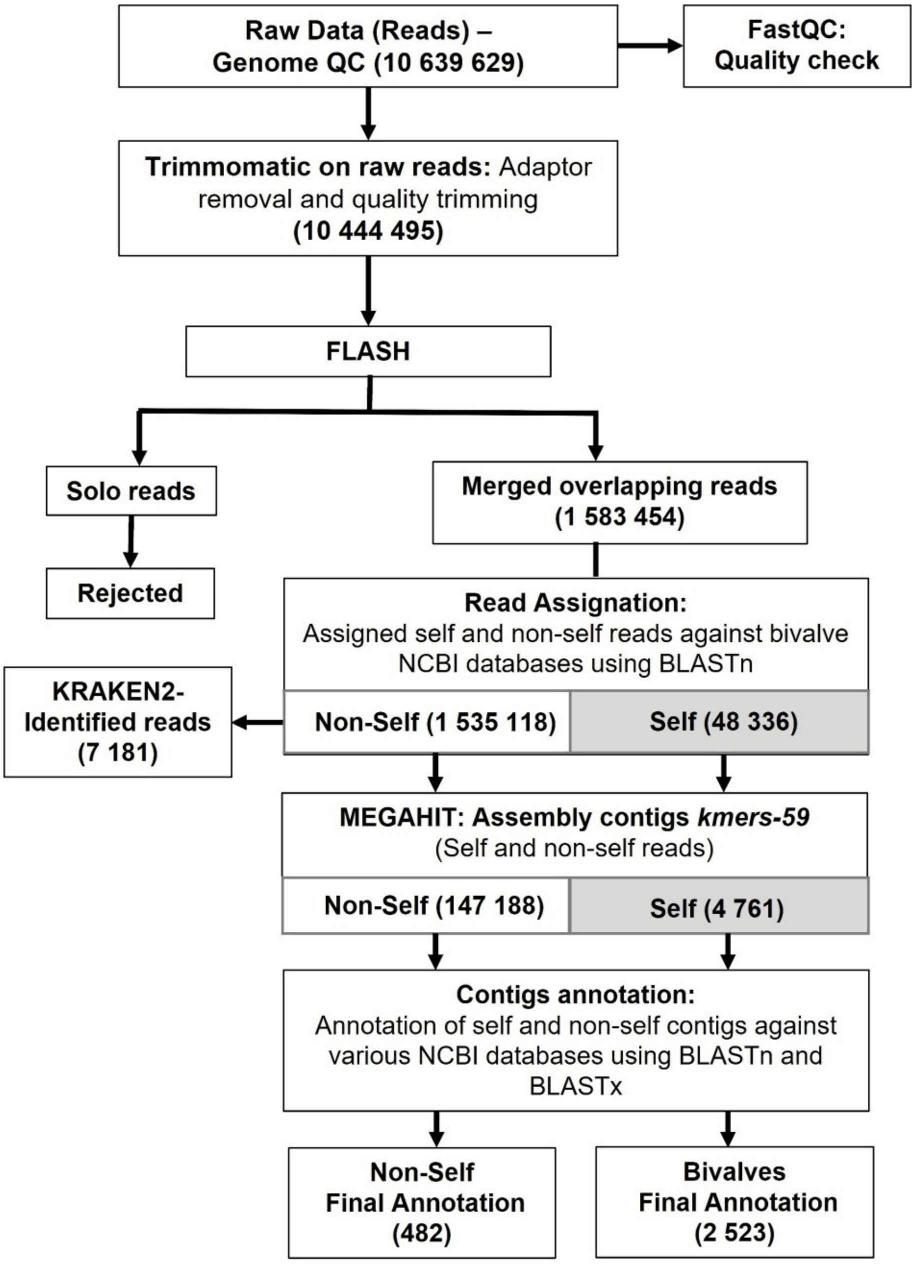

Supp Figure 1. Workflow of bioinformatic analysis of the ccfDNA shotgun sequencing data. Raw data quality was assessed with FastQC . Reads were trimmed with Trimmomatic and merged with FLASH tools. Overlapping reads were then assigned against bivalve databases using BLASTn. Both self and non-self reads were assembled with MEGAHIT. Final contig annotations were obtained using BLASTn and BLASTx with various NCBI databases.

Supp. Figure 2

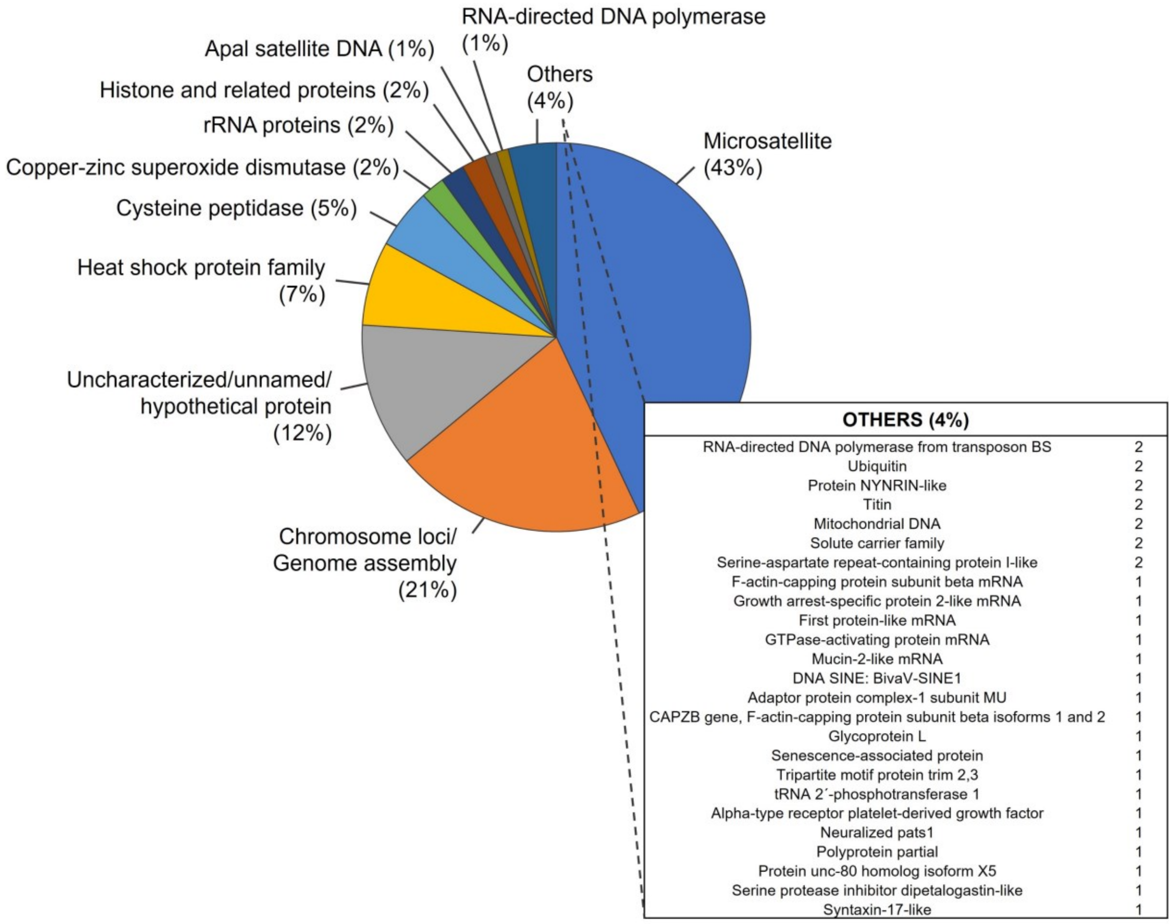

OTHERS (4%)	
RNA-directed DNA polymerase from transposon BS	2
Ubiquitin	2
Protein NYNRIN-like	2
Titin	2
Mitochondrial DNA	2
Solute carrier family	2
Serine-aspartate repeat-containing protein I-like	2
F-actin-capping protein subunit beta mRNA	1
Growth arrest-specific protein 2-like mRNA	1
First protein-like mRNA	1
GTPase-activating protein mRNA	1
Mucin-2-like mRNA	1
DNA SINE: BivaV-SINE1	1
Adaptor protein complex-1 subunit MU	1
CAPZB gene, F-actin-capping protein subunit beta isoforms 1 and 2	1
Glycoprotein L	1
Senescence-associated protein	1
Tripartite motif protein trim 2,3	1
tRNA 2´-phosphotransferase 1	1
Alpha-type receptor platelet-derived growth factor	1
Neuralized pats1	1
Polyprotein partial	1
Protein unc-80 homolog isoform X5	1
Serine protease inhibitor dipetalogastin-like	1
Syntaxin-17-like	1

Supp Figure 2. Detection of DNA fragments of self-origin. Annotation of self assembled contigs (n=580) using BLASTn and BLASTx performed with the Bivalvia libraries (genomes and proteins).

Supp. Figure 3

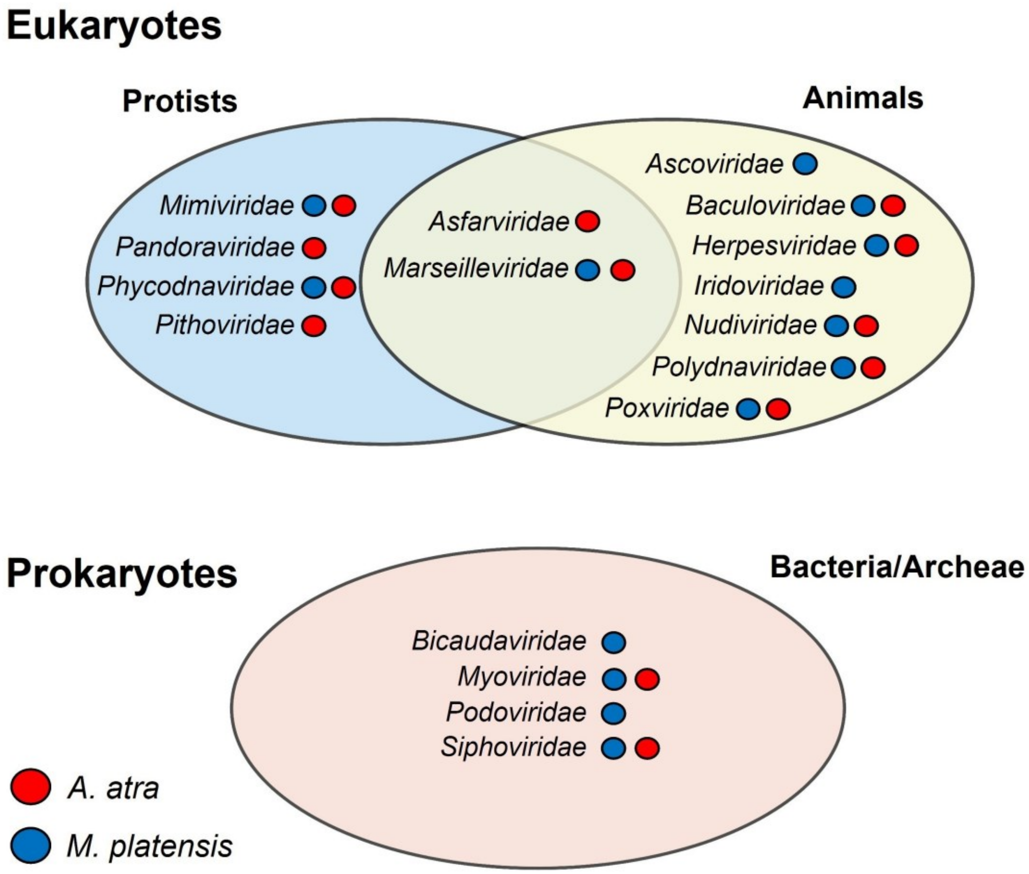

Supp Figure 3. Families of viruses identified in non-self reads for *A. atra* and *M. platensis*. Venn diagram analysis illustrating the the hosts of families of viruses identified and the overlap between *A. atra* (red points) and *M. platensis* (blue points).

Supp. Figure 4

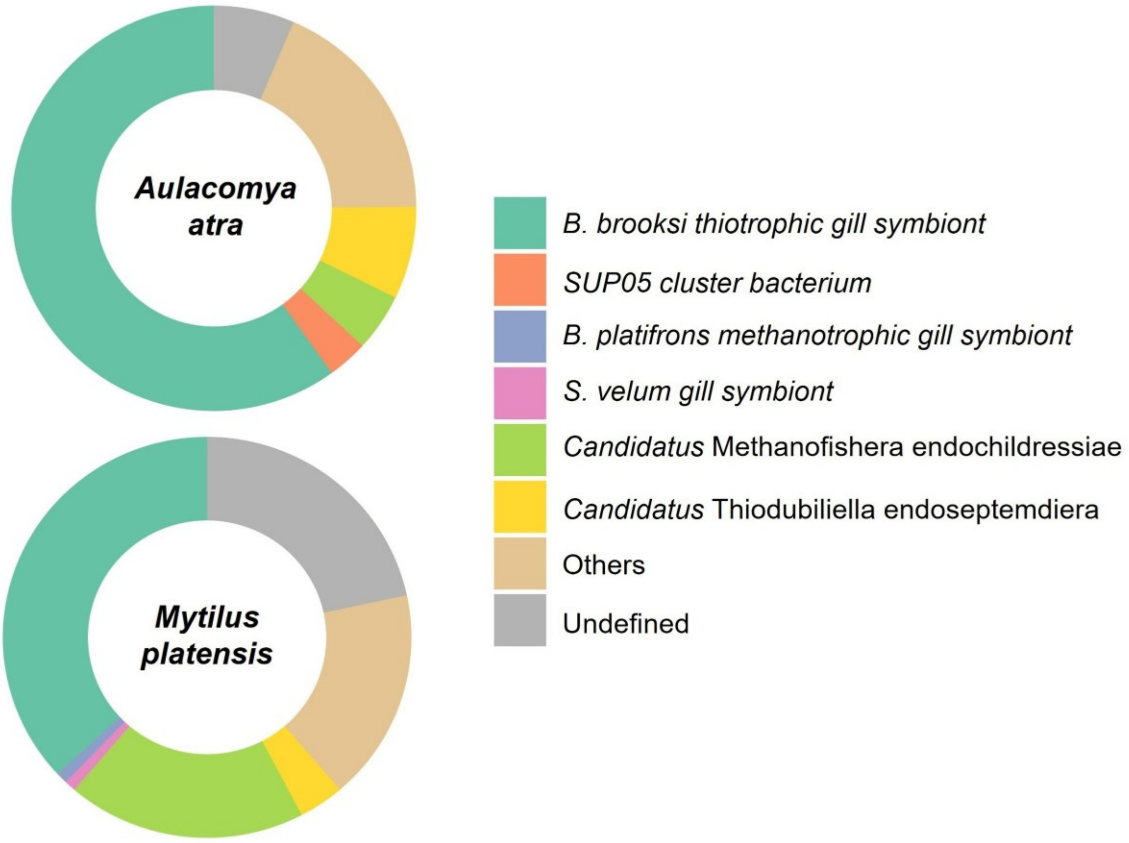

Supp Figure 4. Pie charts showing the composition of the gill-associated bacteria for *A. atra* and *M. platensis*.

Supp. Figure 5

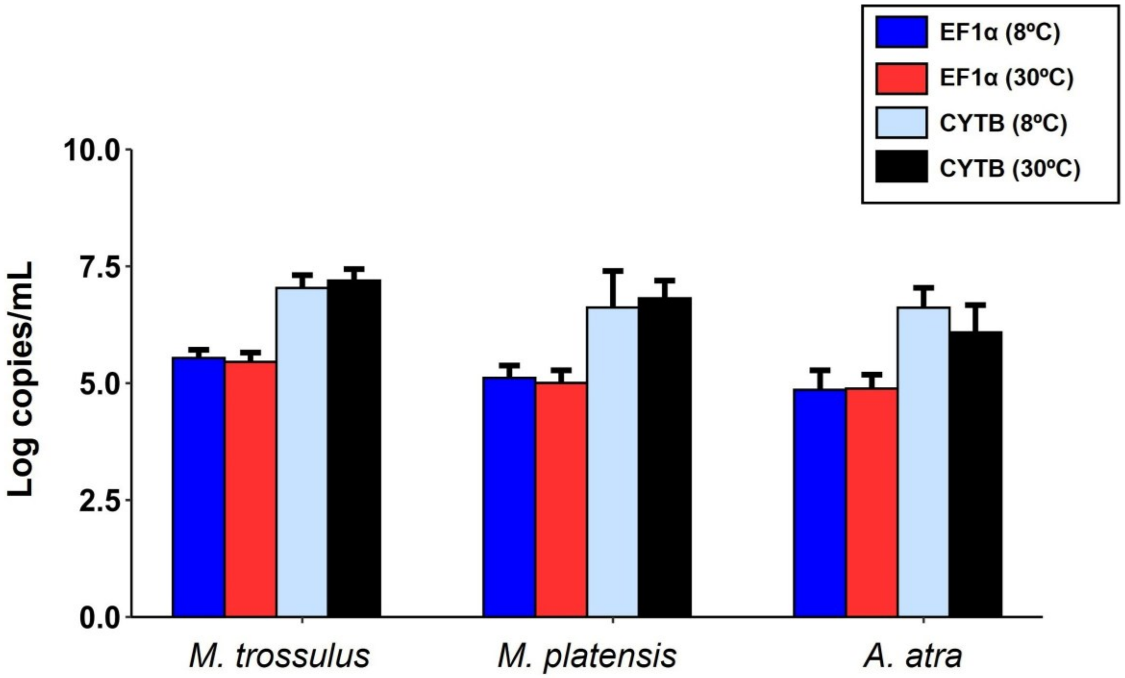

Supp Figure 5. Thermal stress: measure of *EF1α* and CYTB gene levels in ccfDNA of three different *Mythilidae*. Ten mussels of each group (*M.trossulus, M. platensis* and *A. atra)* were placed in sea water at 30°C for 90 minutes or 8°*C* (control group). Hemolymphs were stored on FTA papers and ccfDNA extractions were carried with QIAamp DNA Investigator Kit for *M. platensis* and *A. atra.* Hemolymphs of *M. trossulus* were frozen and extracted with NucleoSnap DNA Plasma Kit. Measures of *EF!a and CYTB genes* by ddPCR amplifications are reported as copies per ml of hemolymph.

Supplementary Table 1. Primers used for different experiments.

Species Target	Gene Target	Primer Name	Primer Sequence (5´ to 3´)	Fragment Size (bp)	Ta† (°C)
			**Droplet digital PCR**		
Mytilus edulis (AY580270.1)	*EF1α*	EF1α835F	CACCACGAGTCTCTCCCAGA	105	53.4 to 63
		EF1α939R	GCTGTCACCACAGACCATTCC		
Mytilus chilensis (KT966847.1)	*CYTB-MC*	Cbmt3828F	GTAGCTTACCTTGGACGGGC	152	51 to 59
		Cbmt3696R	CGGCTAGGGTTGTACTGGTG		
Vibrio spp. (AJ316181.1)	*Vib16S*	Vib550F	GGCGTAAAGCGCATGCAGGT	113	51.9 to 62.3
		Vib663R	GAAATTCTACCCCCCTCTACAG		
Homo sapiens (NM_001042507.4)	*LGALS7*	GAL7HS45F	CAAGTCCTCACTGCCCGAG	188	51.0 to 62.3
		GAL7HS233R	GAGCCTTGCTCCTTGCTGTT		
			**Standard PCR**		
Aulacomya atra (*N/A)	*CYTB-AA*	CbmtAAF	TTAGCTCTGCTTTGCTCGGT	145	60
		CbmtAAR	TCCCAATTCAGGTCAACCCT		
Mytilus edulis (L33448.1)	*18SrRNA*	18SrRNA79F	TAGTGAAACCGCGAATGGCT	296	60
		18SrRNA374R	CCCGTTACCCGTTACAACCA		
Aulacomya atra (Accession : *N/A)	*COX1*	COX1AAF	TACACAGTCCATCCGGTCCC	120	60
		COX1AAR	TCCTGATATGATTTTTCCCCGGT		
Aulacomya atra (*N/A)	*Nd5*	Nd674F	GTTCATTCCCAACATTCGCACA	141	60
		Nd799R	TGTATTTCGGTTGGCTTTCCAC		
Mytilus edulis (AY580270.1)	*EF1α*	EF1α835F	CACCACGAGTCTCTCCCAGA	105	60
		EF1α939R	GCTGTCACCACAGACCATTCC		
Homo sapiens (NM_001042507.4)	*LGALS7*	GAL7HS45F	CAAGTCCTCACTGCCCGAG	188	60
		GAL7HS233R	GAGCCTTGCTCCTTGCTGTT		

*N/A Primers were designed based on the sequence obtained from reads assemblies. PCR amplicons were further obtained and sequenced to validate the presence of mitochondrial DNA.

† Annealing temperature

Supp Table 2. Comparison of ccfDNA extraction methods in hemolymph of *Mythylidae* using two different kits. ccfDNA was extracted from frozen hemolymph or from supernatant spotted on FTA cards using a 5mm punch. NucleoSnap cfDNA kit (Macherey-Nagel, Germany) was used on thawed hemolymph whereas QIAamp DNA Investigator kit (QIAGEN Inc., Canada) could be used dually on both thawed hemolymph or hemolymph preserved on FTA cards.

Species	Extraction kit	n	Punch (mm) or hemolymph (µL)	Concentration (µg) per mL of hemolymph	SDV
M. edulis spp.	Nucleosnap cfDNA	17	1 500 µL	2.67	0.90
M. platensis	QIAamp DNA Investigator	9	5 mm	0.73	0.24
A. atra	QIAamp DNA Investigator	10	5 mm	0.99	0.39
M. edulis spp.	QIAamp DNA Investigator	10	70 µL	7.10	2.03

Supp Table 3. Summary of Kraken2 analysis of non-self reads for *A. atra* and *M. platensis*

Reads	Non-self reads (*A. atra*)	Non-self reads (*M. platensis*)
**Unclassified reads**	**1 558 305**	**1 067 732**
**Classified reads :**	**7 181**	**3 893**
Bacteria	6 719	3 656
Virus	64	57
Archaea	124	96
Unassigned	274	84
